# Interaction effect of serum serotonin level and age on the 12-week pharmacotherapeutic response in patients with depressive disorders

**DOI:** 10.1038/s41598-021-03753-3

**Published:** 2021-12-20

**Authors:** Wonsuk Choi, Ju-Wan Kim, Hee-Ju Kang, Hee Kyung Kim, Ho-Cheol Kang, Ju-Yeon Lee, Sung-Wan Kim, Robert Stewart, Jae-Min Kim

**Affiliations:** 1 Department of Internal Medicine, Chonnam National University Hwasun Hospital, Chonnam National University Medical School, Hwasun, Republic of Korea; 2 Department of Psychiatry, Chonnam National University Medical School, 160 Baekseo-ro, Dong-gu, Gwangju, 61469 Republic of Korea; 3 King’s College London, Institute of Psychiatry, Psychology and Neuroscience, London, UK; 4 South London and Maudsley NHS Foundation Trust, London, UK

**Keywords:** Predictive markers, Outcomes research, Depression

## Abstract

Despite the recognized antidepressant role of serotonin (5-hydroxytryptamine [5-HT]) signaling pathways in the central nervous system, the association between baseline peripheral 5-HT level and the antidepressant treatment response in clinical studies remains debatable. We investigated the interaction effects of baseline serum 5-HT level and age on the 12-week remission in outpatients with depressive disorders who received stepwise antidepressant treatment. Baseline serum serotonin levels were measured and the age of 1094 patients recorded. The patients received initial antidepressant monotherapy; then, patients with an insufficient response or who experienced uncomfortable side effects received alternative treatments every 3 weeks (3, 6, and 9 weeks). Subsequently, 12-week remission, defined as a Hamilton Depression Rating Scale (HAMD) score of ≤ 7, was evaluated. Individual and interaction effects of serum 5-HT level (as a binary [low *vs*. high, based on the median value of 72.6 ng/mL] or continuous variable) and age (as a binary [< 60 *vs*. ≥ 60 years] or continuous variable) on the 12-week remission rate were analyzed using logistic regression models after adjusting for relevant covariates. High 5-HT (≥ 72.6 ng/mL) and age ≥ 60 years were associated with the highest 12-week remission rates and a significant multiplicative interaction effect. The interaction effect of the two variables on the 12-week remission rate was significant even when analyzed as a continuous variable. Our study suggests that the association between baseline serum 5-HT level and 12-week antidepressant treatment outcomes differs according to patient age.

## Introduction

Depressive disorders are a significant contributor to worldwide disability, affecting more than 250 million people^1,2^. Antidepressants are the first-line treatment for moderate to severe depressive disorders. Currently used antidepressants have similar efficacy and lead to remission in less than one-third of patients after initial antidepressant trials^3,4^. Considering the high disease burden and low initial antidepressant treatment response, developing biomarkers that predict the efficacy of antidepressants could aid in determining individualized treatment strategies for depressive disorders. However, clinically meaningful biomarkers that predict antidepressant efficacy have not yet been developed^5^.

Serotonin (5-hydroxytryptamine [5-HT]) is a monoamine neurotransmitter synthesized from the essential amino acid tryptophan that mainly exerts its biological action by binding to 5-HT receptors (HTRs). In the central nervous system (CNS), 5-HT has pleiotropic functions in the regulation of mood^6^, sleep–wake behavior^7^, and appetite^8^. In previous studies, decreased 5-HT metabolite levels in the cerebrospinal fluid^9^ or attenuated HTR signaling in the CNS^10–12^ were observed in patients with depressive disorders. Although the association between peripheral 5-HT and central 5-HT remains unclear^13,14^, peripheral 5-HT levels have been investigated as a predictor of antidepressant treatment response due to the suggested role of attenuated serotonergic neurotransmission in the pathogenesis of depression. However, inconsistent results were reported in previous studies regarding associations between peripheral 5-HT levels and antidepressant treatment outcomes. In several studies, higher 5-HT levels were reportedly associated with a better treatment response^15,16^; however, association with a worse treatment response^17,18^, or no significant association^19^ has been reported in other studies.

Depressive disorders are frequent in the elderly population^20^. Because serotonergic neurotransmission, especially HTR1A^21–23^ and HTR2A^24–27^ signalling, is downregulated in older individuals, the causal effect of 5-HT level on depression treatment outcomes may be larger in older than younger individuals, but this has not been studied to date.

The objective of the present study was to investigate the interaction between baseline 5-HT level and age on the 12-week remission rate in a prospective cohort of Korean patients with depressive disorders receiving stepwise antidepressant treatment.

## Results

### Recruitment and treatment flow

**Figure 1:**
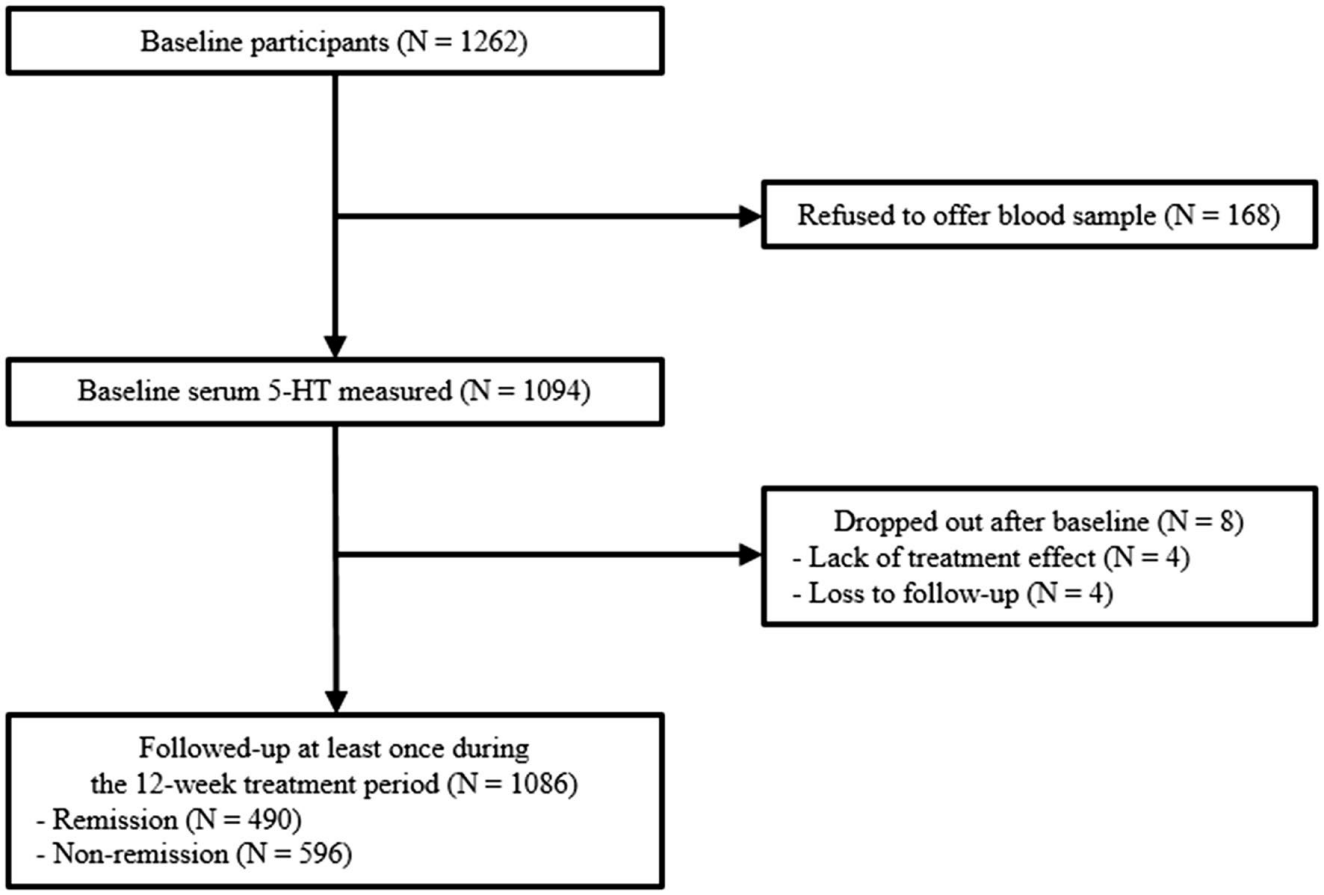
Participant recruitment and flow.

Patient flow over the 12-week period is shown in Fig. 1. Among 1262 patients evaluated at baseline, serum 5-HT levels were measured in 1094 (86.7%), and 1086 (86.1%) were followed up at least once during the 12-week treatment period. Reasons for drop-out were a lack of treatment effect (N = 4) and loss to follow-up (N = 4). Among the 1086 patients, 490 (45.1%) achieved 12-week remission.

### Baseline characteristics based on 5-HT levels

Among 1086 participants, the median (interquartile range (IQR)) and mean (standard deviation (SD)) serum 5-HT values were 72.6 (67.1) and 79.8 (52.6) ng/mL, respectively. The age range, median (IQR), and mean (SD) were 17–85, 59.0 (19.0), and 56.9 (14.9) years, respectively. The number of participants < 60 years of age was 567 (52.2%), and 519 (47.8%) were ≥ 60 years of age. The age range, median (IQR), and mean (SD) of the participants aged < 60 years were 17–59, 50.0 (17.0), and 50.0 (11.6) years, respectively, while those of participants aged ≥ 60 years were 60–85, 68.0 (10.0), and 69.0 (6.1) years, respectively. Among the 1086 study participants, the baseline serum 5-HT level and age had a significant negative correlation (r^2^ =  − 0.168, p < 0.001).

Baseline characteristics compared based on the median serum 5-HT value (72.6 ng/mL) in patients who underwent up to 12 weeks of treatment are summarized in Table 1. A higher 5-HT level was significantly associated with a younger age, higher educational level, lower frequency of low monthly income, lower frequency of melancholic features, higher frequency of atypical features, younger age at onset, and higher Social and Occupational Functioning Assessment Scale (SOFAS) score. Baseline characteristics compared based on age (< 60 *vs*. ≥ 60 years) in patients who underwent up to 12 weeks of treatment are summarized in Supplementary Table  1. Age ≥ 60 years was significantly associated with a lower educational level, higher frequency of living alone, higher frequency of religious observance, higher frequency of low monthly income, higher frequency of melancholic features, lower frequency of atypical features, younger age at onset, longer duration of illness, lower frequency of recurrent depression, lower number of depressive episodes, lower frequency of family history of depression, lower frequency of history of suicide attempt, higher number of physical disorders, and lower Hospital Anxiety Depression Scale-anxiety subscale (HADS-A) score. Based on the causal system that generated the data and potential collinearity among the variables, 10 variables (educational level, living alone, religious observance, melancholic features, atypical features, age at onset, duration of present episode, HAMD score, HADS-A score, and SOFAS score) were included as covariates in the adjusted analyses.

**Table 1:** Baseline characteristics based on serum serotonin levels in patients with depressive disorders.

	Up to 12-week treatment (N = 1086)			
	Low-5-HT (N = 543)	High-5-HT (N = 543)	Statistic coefficients^a^	P-value
Age, mean (SD) years	59.2 (14.2)	54.7 (15.3)	t = 5.066	** < 0.001**^**b**^
Gender, N (%) female	375 (69.1)	370 (68.1)	χ^2^ = 0.107	0.744
Education, mean (SD) years	8.5 (4.8)	9.7 (4.7)	t = − 3.887	** < 0.001**^**b**^
Marital status, N (%) unmarried	161 (29.7)	165 (30.4)	χ^2^ = 0.070	0.791
Living alone, N (%)	93 (17.1)	74 (13.6)	χ^2^ = 2.554	0.110
Religious observance, N (%)	310 (57.1)	297 (54.7)	χ^2^ = 0.631	0.427
Unemployed status, N (%)	170 (31.3)	146 (26.9)	χ^2^ = 2.571	0.109
Monthly income, N (%) < 2,000 USD	342 (63.0)	306 (56.4)	χ^2^ = 4.959	**0.026**
Body mass index, mean (SD) kg/m^2^	23.0 (3.2)	23.4 (3.2)	t = − 1.869	0.062
Major depressive disorder, N (%)	465 (85.6)	460 (84.7)	χ^2^ = 0.182	0.669
Melancholic feature, N (%)	93 (17.1)	69 (12.7)	χ^2^ = 4.179	**0.041**
Atypical feature, N (%)	26 (4.8)	43 (7.9)	χ^2^ = 4.473	**0.034**
Age at onset, mean (SD) years	1.6 (0.5)	1.5 (0.5)	t = 2.983	**0.003**
Duration of illness, mean (SD) years	5.5 (9.9)	4.6 (8.1)	t = 1.659	0.097
Recurrent depression, N (%)	284 (52.3)	286 (52.7)	χ^2^ = 0.015	0.903
Number of depressive episodes, mean (SD)	1.1 (1.4)	1.1 (1.5)	t = − 0.412	0.680
Duration of present episode, mean (SD) months	7.9 (11.7)	6.9 (8.9)	t = 1.466	0.143
Family history of depression, N (%)	71 (13.1)	87 (16.0)	χ^2^ = 1.896	0.169
History of suicide attempt, N (%)	54 (9.9)	41 (7.6)	χ^2^ = 1.949	0.163
Number of physical disorders, mean (SD)	1.5 (0.5)	1.5 (0.5)	t = 1.457	0.145
Hamilton Depression Rating Scale	20.7 (4.1)	20.7 (4.2)	t = 0.007	0.994
Hospital Anxiety and Depression Scale-anxiety subscale	11.9 (3.9)	11.8 (4.2)	t = 0.382	0.702
EuroQol-5D	8.9 (1.5)	8.9 (1.5)	t = − 0.060	0.952
Social and Occupational Functional Assessment Scale	55.4 (7.4)	56.5 (7.5)	t = − 2.281	**0.023**

Significant values are in bold.

^a^Independent two sample *t*-test or χ^2^ test, as appropriate.

^b^Values show statistical significance after Bonferroni correction.

### Effects of serum 5-HT level and age on 12-week remission status

**Figure 2:**
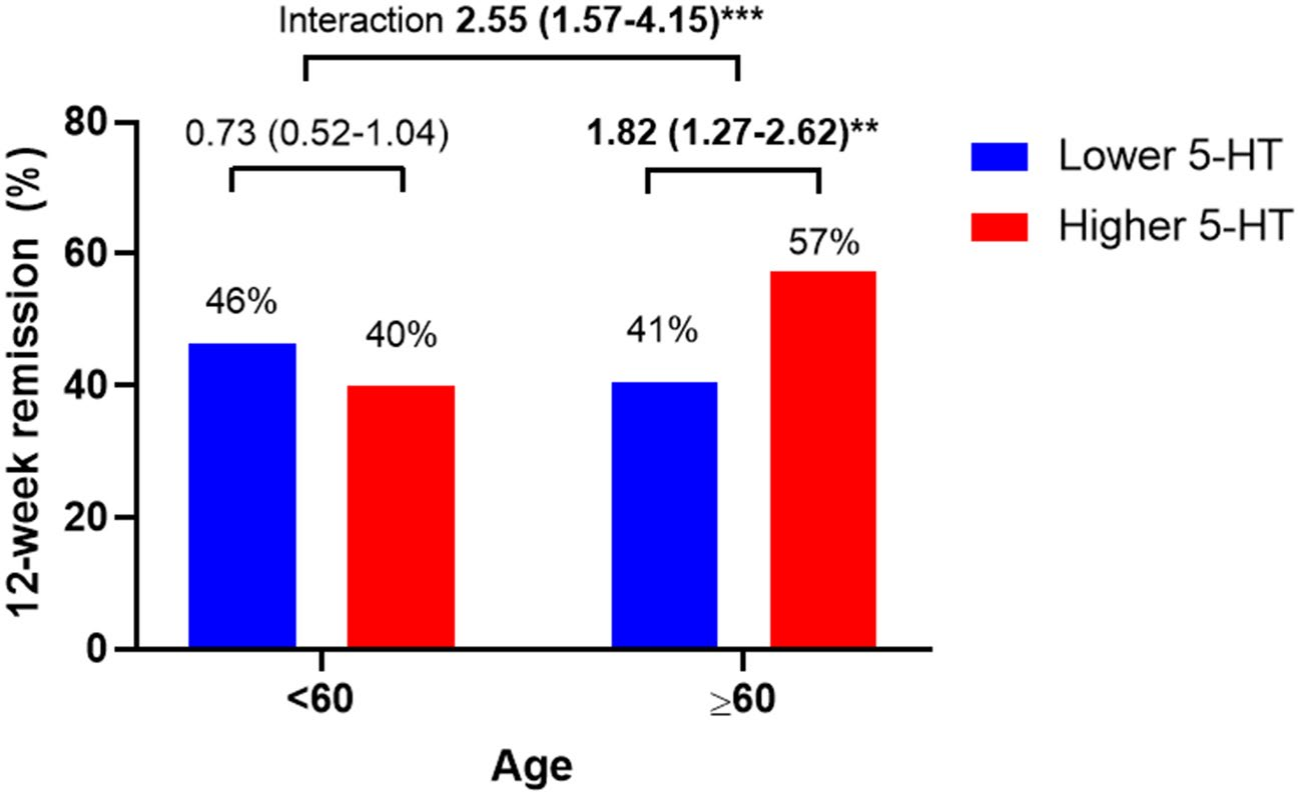
The 12-week remission rates according to baseline serum 5-HT level and age. Data are presented as odds ratios (95% confidence intervals) adjusted for educational level, living alone, religious observance, melancholic features, atypical features, age at onset, duration of present episode, HAMD score, HADS-A score, and SOFAS score. **P < 0.01; ***P < 0.001.

The individual effects of baseline serum 5-HT level and age on 12-week remission are shown in Table 2. As a binary variable, a high baseline serum 5-HT level was not significantly associated with the 12-week remission rate, whereas as a continuous variable, high baseline 5-HT was significantly associated with the 12-week remission rate in adjusted analyses. Greater age, as a binary or continuous variable, was not associated with the 12-week remission rate in the adjusted analyses. The interaction effect of baseline serum 5-HT level and age is shown in Fig. 2 and Supplementary Table 2. As a binary variable, a high baseline serum 5-HT level was significantly associated with the 12-week remission rate only in participants aged ≥ 60 years. The interaction effect on the 12-week remission rate was statistically significant after adjusting for the relevant covariates (odds ratio [OR] = 2.55; 95% confidence intervals [CI] = (1.57–4.15); P < 0.001). The interaction effect of baseline serum 5-HT level and age, analyzed as continuous variables, on the 12-week remission rate was also significant (OR = 1.15; 95% CI = 1.06–1.25); P = 0.001).

**Table 2:** Individual effects of baseline serum 5-HT level and age on 12-week remission.

Exposure	Group	N	12-week remission		
			No. (%) presence	OR (95% CI)	
				Unadjusted	Adjusted^a^
Serum 5-HT	Lower	543	234 (43.1)	1.00	1.00
	Higher	543	256 (47.1)	1.18 (0.93–1.50)	1.14 (0.89–1.46)
Serum 5-HT (increasing)	N/A	1086	N/A	1.14 (1.01–1.28)*	1.14 (1.01–1.30)*
Age	< 60	567	242 (42.7)	1.00	1.00
	≥ 60	519	248 (47.8)	1.23 (0.97–1.56)	1.02 (0.73–1.44)
Age (increasing)	N/A	1086	N/A	1.11 (1.03–1.21)**	1.13 (0.96–1.33)

Significant values are in bold.

^a^Adjusted for education, living alone, religious observance, melancholic features, atypical features, age at onset, duration of present episode, HAMD, HADS-A, and SOFAS. *P < 0.05; **P < 0.01.

### Effects based on initial antidepressant type

The comparison of baseline characteristics based on initial antidepressant type in patients who underwent up to 12 weeks of treatment is shown in Supplementary Table 3. The selective 5-HT reuptake inhibitor (SSRI) group was significantly associated with lower Hamilton Depression Rating Scale (HAMD) score, lower EuroQol-5D (EQ-5D) score, and higher SOFAS score. A comparison of the 12-week remission rate, serum 5-HT level, and age between the initial antidepressant types is shown in Supplementary Table 4. There was no difference in the 12-week remission rate or serum 5-HT level between the SSRI and non-SSRI groups. However, mean age was lower in the SSRI than non-SSRI group, although there was no difference in median age between the two groups. The individual effects of baseline serum 5-HT level and age on the 12-week remission rate based on the initial antidepressant type are shown in Supplementary Table 5. Among 672 patients who initially received SSRIs, the baseline serum 5-HT level and age, as both binary and continuous variables, were not significantly associated with the 12-week remission rate in the adjusted analyses. Similarly, among 414 patients who initially received non-SSRIs, the baseline serum 5-HT level and age, as both binary and continuous variables, were not significantly associated with the 12-week remission rates in adjusted analyses. The interaction effect of baseline serum 5-HT level and age based on the initial antidepressant type is shown in Supplementary Fig. 1 and Supplementary Table 6. As a binary variable, high baseline serum 5-HT level was significantly associated with the 12-week remission rate in individuals aged ≥ 60 years, regardless of the initial antidepressant type. The interaction effect on the 12-week remission rate was statistically significant after adjusting for the relevant covariates in both the SSRI (OR = 2.34; 95% CI = 1.26–4.36; p = 0.007) and non-SSRI (OR = 3.05; 95% CI = 1.38–6.73; p = 0.006) groups. However, analyzed as continuous variables, the interaction effect on the 12-week remission rate was statistically significant only in the SSRI group after adjusting for the relevant covariates (OR = 1.19; 95% CI = 1.07–1.32; P = 0.001).

## Discussion

In the present study, we used data from a naturalistic prospective pharmacotherapeutic study, which reflects real-world clinical practice. A high 5-HT level and age ≥ 60 years were associated with the highest 12-week remission rate and a significant multiplicative interaction effect, which remained significant even after adjustment for the relevant covariates. The interaction effect between the two variables on the 12-week remission rate was significant even when analyzed as a continuous variable.

As stated in the “Introduction”, the associations between peripheral (plasma, serum, and platelet) 5-HT levels and antidepressant treatment outcomes have been inconsistent in previous clinical studies. Reportedly, high baseline plasma 5-HT levels were associated with better 4- and 8-week SSRI responses in several studies^15,16^, indicating that treatment-related changes in plasma 5-HT levels, as well as baseline plasma 5-HT levels, are associated with a better SSRI response. However, in other studies, high baseline platelet 5-HT levels were associated with worse 4- and 6-week SSRI responses^17,18^. In another study, baseline serum or plasma 5-HT levels exhibited no association with the 4-week SSRI response^19^. The discrepancies in the results of previous studies do not appear to be due to differences in study design. The treatment response was assessed around 4 to 8 weeks in all studies. Furthermore, all studies included patients with major depressive disorders. Although the types of SSRIs prescribed were different, inconsistent results were also found in two studies with similar study designs in which the predictive effect of pre-treatment plasma 5-HT levels for the 4-week SSRI response was evaluated^15,19^.

In previous studies in which the predictive effect of baseline 5-HT levels for antidepressant treatment responses was investigated, the interaction between 5-HT levels and other variables was not considered. This may be the reason for the conflicting results regarding the association between baseline peripheral 5-HT levels and future antidepressant treatment responses. The present study results showing that a higher serum 5-HT level predicted a better treatment response only in patients ≥ 60 years of age may provide clues for addressing the current controversy. Synergistic effects between high 5-HT levels and age ≥ 60 years in our cohort are biologically plausible. Decreased central HTR binding activity, especially in HTR1A^11,12,28^ and HTR2A^10,29^, measured using positron emission tomography have been reported in patients with depressive disorders. Because HTR1A^21–23^ and HTR2A^24–27^ binding capacities are downregulated in older individuals, high 5-HT levels may be predictive of 12-week remission only in subjects ≥ 60 years of age due to decreased 5-HT signaling activity in the elderly.

Unlike in previous studies in which the association between 5-HT levels and antidepressant treatment outcomes in patients who only received SSRIs was analyzed^15–19^, we included patients who received various types of antidepressants. When 5-HT and age were analyzed as binary variables, a multiplicative interaction effect of high 5-HT level and age ≥ 60 years on the 12-week remission rate was observed in patients who received initial SSRI or non-SSRI treatment. One potential mechanism underlying this observation is the change in tryptophan metabolism due to systemic inflammation, which shifts tryptophan metabolism from the 5-HT pathway to the kynurenine pathway by inducing the activity of indoleamine-2,3-dioxygenase^30^. Because increased inflammation is associated with a worse antidepressant response regardless of antidepressant type^31^, low 5-HT levels may be indicative of high inflammatory activity and therefore predict worse antidepressant responses regardless of antidepressant type. Another potential explanation for our result is decreased tryptophan availability. Tryptophan depletion reportedly decreases the mood in remitted major depressive disorder (MDD) patients regardless of the use of antidepressants^32^. Because a decreased mood can be a predictor of a worse antidepressant response^33^ and peripheral 5-HT levels are determined in part by tryptophan availability, we hypothesize that a lower 5-HT level may predict a worse antidepressant response by acting as a surrogate of low tryptophan availability regardless of antidepressant type. However, the interaction effect of a high 5-HT level and old age on the 12-week remission rate was not observed when 5-HT and age were analyzed as continuous variables in patients who initially received non-SSRI treatment. Since the interaction effect of 5-HT level and age on the 12-week remission rate in the non-SSRI group differed depending on the analysis method, future prospective studies with a greater number of patients are required to further explore this issue.

Several issues should be considered. First, because 5-HT cannot cross the blood–brain barrier, the peripheral and central 5-HT systems are functionally separated, and the correlation between peripheral and central 5-HT levels is controversial^13,14,34^. Further studies are needed to determine the detailed mechanisms by which peripheral 5-HT levels affect the antidepressant response in patients with depressive disorders. Second, we measured 5-HT levels in serum rather than in plasma or platelets. Peripheral 5-HT is mainly synthesized by enterochromaffin cells in the gut^35^. Once released from the gut, most 5-HT is taken up into platelets (> 95%), and the remaining free 5-HT level in the circulation is very low^36–38^. Therefore, plasma 5-HT levels reflect the bioactive free 5-HT, platelet 5-HT levels reflect the major 5-HT pool in the periphery, and serum 5-HT levels reflect both the bioactive 5-HT and 5-HT pools^38,39^. In the present study, we showed that the serum 5-HT level can be used as a biomarker of the antidepressant response. Based on these results, we hypothesize that both the bioactive 5-HT and 5-HT pools in the periphery may be predictive of antidepressant treatment outcomes in patients ≥ 60 years of age. To verify this hypothesis, the interaction effect of plasma and/or platelet 5-HT levels and age ≥ 60 years on 12-week remission should be investigated in patients with depressive disorders.

The strength of this study was the large sample size, and participants were evaluated using a structured research protocol and standardized scales. As stated above, this is the first study in which the interaction effect of peripheral 5-HT level and age on antidepressant treatment outcome was reported. Furthermore, because this was a naturalistic prospective study, which reflects actual clinical practice situations, results obtained in this study can serve as a basis for developing biomarkers of antidepressant treatment response in real-world clinical practice.

The present study had several limitations. First, the association between treatment-related changes in serum 5-HT levels and treatment outcomes could not be assessed because longitudinal data on serum 5-HT levels were lacking. Second, because the study was naturalistic in design, treatment was based on patient preferences under the guidance of a physician rather than determined based on a preset protocol; therefore, inter-physician variability might have affected the outcomes. However, because physicians guided treatment decisions without knowing the baseline 5-HT levels, inter-physician variability likely did not affect the outcomes. Third, because various antidepressants were initially used and different treatment strategies (S, A, C, and mixtures of these approaches) were implemented from 3 weeks after the start of antidepressant monotherapy, too many variables existed for evaluating the interaction effect of 5-HT level and age on the antidepressant response based on the type of antidepressant. Fourth, the reliability of 5-HT measurements was not evaluated. However, we used a kit with well-established validity, manufactured by the Recipe (Munich, Germany).

Despite the recognized antidepressant role of 5-HT/HTR signaling pathways in the CNS, the association between baseline peripheral 5-HT level and the antidepressant treatment response in clinical studies remains debatable. In the present study, we investigated the interaction between baseline 5-HT level and age on the 12-week remission rate in patients with depressive disorders who received stepwise antidepressant therapy. Our study suggests that the association between baseline serum 5-HT level and 12-week antidepressant treatment outcomes is strengthened by accounting for patient age. Because this was a non-randomized trial, the interaction effect of serum 5-HT level and age on treatment outcome in patients receiving antidepressants should be evaluated in future randomized trials.

## Materials and methods

### Study outline

This study was carried out as a component of the MAKE Biomarker discovery for Enhancing antidepressant Treatment Effect and Response (MAKE BETTER) program. Details of the study have been published as a design paper^40^ and registered with cris.nih.go.kr (identifier: KCT0001332, registration date: 30/12/2014). Data on socio-demographic and clinical characteristics were obtained using a structured clinical report form (CRF) by clinical research coordinators, who were blind to treatment modalities. They were trained in CRF implementation and data collection methods by the research psychiatrists. Patients’ data were recorded on a CRF, registered via the website of the MAKE BETTER study (http://icreat.nih.go.kr/icreat/webapps/com/hismainweb/jsp/cdc_n2.live) within 3 days, and monitored by data management center personnel. This study was approved by the Chonnam National University Hospital Institutional Review Board (CNUH 2012-014).

### Participants

Patients with depressive disorders were recruited from March 2012 to April 2017 who had attended the outpatient psychiatric department of Chonnam National University Hospital. All inclusion instances represented new treatment episodes—i.e. taking newly initiated antidepressant treatment—whether depressive symptoms were first-onset or recurrent. Because the primary aim of the MAKE BETTER STUDY was to identify predictive markers for antidepressant treatment outcomes, all participants who provided consent received only antidepressants. Research psychiatrists assessed and diagnosed depressive disorders using the Mini-International Neuropsychiatric Interview (MINI)^41^, a structured diagnostic psychiatric interview based on the Diagnostic and Statistical Manual of Mental Disorders, Fourth Edition (DSM-IV) criteria. As the aim of the study was to reflect a real-world clinical setting as closely as possible, broad inclusion criteria and minimal exclusion criteria were applied. Inclusion criteria were: (i) aged older than 7 years; (ii) diagnosed with MDD, dysthymic disorder, or depressive disorder not otherwise specified (NOS); (iii) HAMD score ≥ 14^42^; (iv) able to complete questionnaires, understand the objective of the study, and sign the informed consent form. Exclusion criteria were as follows: (i) unstable or uncontrolled medical condition; (ii) unable to complete the psychiatric assessment or comply with the medication regimen, due to a severe physical illness; (iii) current or lifetime DSM-IV diagnosis of bipolar disorder, schizophrenia, schizoaffective disorder, schizophreniform disorder, psychotic disorder NOS, or other psychotic disorder; (iv) history of organic psychosis, epilepsy, or seizure disorder; (v) history of anticonvulsant treatment; (vi) hospitalization for any psychiatric diagnosis apart from depressive disorder (e.g., alcohol/drug dependence); (vii) electroconvulsive therapy received for the current depressive episode; (viii) pregnant or breastfeeding. All participants reviewed the consent form and written informed consent was obtained. For participants aged under 16, written consent was obtained from a parent or legal guardian, and written assent was obtained from the participant.

### Primary measures

#### Serum 5-HT

Participants were instructed to fast (except water) starting the previous night for blood sampling. The participants were asked to sit quietly and relax for 25–45 min before blood samples were obtained. Serum 5-HT levels were measured using a ClinRep high-performance liquid chromatography kit (Recipe, Munich, Germany) at the Global Clinical Central Lab (Yongin, Korea). Patients were divided based on the median 5-HT level into low- and high-5-HT groups.

#### Age

Information on age at baseline was collected. To determine the effect of baseline 5-HT level on the response to antidepressant treatment in late-life depression, subjects were divided into groups comprising those < 60 years of age and those ≥ 60 years of age following previous study protocols^43,44^.

### Baseline covariates

Socio-demographic characteristics obtained comprised gender, years of formal education, marital status (currently married or not), cohabiting status (living alone or not), religion (religious observation or not), occupation (current employed status or not), income (above or below 2000 USD), and body mass index (BMI). Clinical characteristics evaluated comprised diagnoses of depressive disorders as mentioned above with certain specifiers, age at onset and duration of illnesses, history of previous depressive episodes (recurrent or first episode), number of previous depressive episodes, duration of present episode, family history of depression, history of suicide attempt, and number of concurrent physical disorders (applying a questionnaire enquiring about 15 different systems or disorders). Assessment scales for investigating symptoms and function were administered. Depressive symptoms were evaluated by the HAMD, anxiety symptoms by the HADS-A^45^, quality of life by the EQ-5D^46^, and functioning levels by the SOFAS.

### Pharmacotherapy

Details of the treatment in this study have been previously published^40,47^. Before the start of treatment, a comprehensive review was made of patient clinical manifestations (e.g., psychotic and anxiety symptoms), the severity of illness, physical comorbidity and medication profiles, and any history of previous treatments. Minimal and maximal dosages of pharmacological agents were determined based on existing treatment guidelines^48,49^. In the first treatment (Step 1), patients received antidepressants for 3 weeks after taking into consideration the data and treatment guidelines^49–51^. The antidepressants used included bupropion, desvenlafaxine, duloxetine, escitalopram, fluoxetine, mirtazapine, paroxetine, sertraline, venlafaxine, and vortioxetine. Initially prescribed antidepressants were classified as SSRIs; escitalopram, sertraline, paroxetine, fluoxetine or non-SSRIs, which included a noradrenergic and specific 5-HT antidepressant (mirtazapine), 5-HT-norepinephrine reuptake inhibitors (venlafaxine, duloxetine, desvenlafaxine), a norepinephrine-dopamine reuptake inhibitor (bupropion), and a 5-HT modulator and stimulator (vortioxetine). After Step 1 antidepressant monotherapy, next-step pharmacotherapy was administered every 3 weeks (3, 6, and 9 weeks with a 3-day allowable window) whenever needed. At the end of each step, overall effectiveness and tolerability were reviewed to proceed with measurement-based next-step treatments. In cases of insufficient improvement (HAMD score reduction of < 30% from baseline) or intolerable side effects, patients were instructed to choose whether they would prefer to remain in the current treatment step or proceed to next-step strategies consisting of switching (S), augmentation (A), and/or combination (C), with S + A, S + C, A + C, or S + A + C treatments possible. A HAMD score reduction of < 30% from baseline over 3 weeks was adopted as the threshold because in most previous studies, early non-improvement was defined as HAMD score reduction < 20% over 2 weeks^52–55^. Patients were also allowed to receive next-step treatment if they exhibited sufficient improvement (HAMD score reduction ≥ 30% from baseline) and side effects were absent/tolerable. For determining treatment strategies, each patient’s preference was given priority to maximize medication compliance and treatment outcomes^56^. S and C antidepressants included bupropion, desvenlafaxine, duloxetine, escitalopram, fluoxetine, mirtazapine, paroxetine, sertraline, venlafaxine, and vortioxetine. A drugs included buspirone, lithium, and triiodothyronine, as well as the atypical antipsychotics aripiprazole, risperidone, olanzapine, quetiapine, and ziprasidone.

### Outcome

Remission was defined as a HAMD score ≤ 7. Remission at 12 weeks was used in order to investigate the individual and interactive effects of baseline serum 5-HT levels and age on the antidepressant treatment outcomes.

### Statistical analysis

Patients baseline data were compared based on 5-HT level, age, and initial antidepressant type after up to 12 weeks of treatment using independent *t*-tests or chi-square tests. Covariates for the adjusted analyses were selected based on the causal system that generated the data and potential collinearity among the variables. The 12-week remission rate, 5-HT level, and age were compared between the initial antidepressant types using the Chi-square or Mann–Whitney U test. The correlation between baseline serum 5-HT level and age was analyzed by Spearman rank-order correlation analysis. The individual and interaction effects of serum 5-HT level (as a binary [low *vs*. high] or continuous variable) and age (as a binary [< 60 *vs*. ≥ 60 years] or continuous variable) on the 12-week remission rate were analyzed using binominal logistic regression, before and after adjusting for potential covariates, in all three groups (all subjects, subjects who initially received SSRIs, and subjects who initially received non-SSRIs). Serum 5-HT level and age were rescaled by z scores and age per decade, respectively, when analyzed as continuous variables in logistic regression model. All statistical tests were two-sided and a P-value < 0.05 considered to indicate statistical significance. Bonferroni correction was used to maintain an overall type 1 error rate of 0.05 for 24 comparisons of the baseline characteristics. A two-sided P-value of 0.002 (0.05/24) was taken to indicate statistical significance. Statistical analyses were performed using IBM SPSS Statistics (version 25).

### Statement of ethics

All patients gave written informed consent to participate in the study and use their data. The study was conducted in accordance with the Helsinki Declaration of 1975, as revised in 2008 and approved by the Ethics Commission of the Chonnam National University Hospital Institutional Review Board (CNUH 2012-014) as it uses de-identified data. It was registered at cris.nih.go.kr (identifier: KCT0001332).

## Supplementary Information

Supplementary Information.
