# Supplementary material for: Interaction effect of serum serotonin level and age on the 12-week pharmacotherapeutic response in patients with depressive disorders
Source: Sci Rep. 2021 Dec 20;11:24226. doi: 10.1038/s41598-021-03753-3 (PMC8688427; doi:10.1038/s41598-021-03753-3)
Supplement: Supplementary file 1 — Supplementary Information. [file 41598_2021_3753_MOESM1_ESM.docx]

**Supplementary Information**

**Interaction effect of serum serotonin level and age on the 12-week pharmacotherapeutic response in patients with depressive disorders**

Wonsuk Choi^a^, Ju-Wan Kim^b^, Hee-Ju Kang^b^, Hee Kyung Kim^a^, Ho-Cheol Kang^a^, Ju-Yeon Lee^b^, Sung-Wan Kim^b^, Robert Stewart^c,d^, and Jae-Min Kim^b^

^a^Department of Internal Medicine, Chonnam National University Hwasun Hospital, Chonnam National University Medical School, Hwasun, Korea, ^b^Department of Psychiatry, Chonnam National University Medical School, Gwangju, Korea, ^c^ King’s College London, Institute of Psychiatry, Psychology and Neuroscience, London, UK, ^d^ South London and Maudsley NHS Foundation Trust, London, UK

| **Supplementary Table 1.**  Baseline characteristics based on age in patients with depressive disorders | | | | | | | |  |
| --- | --- | --- | --- | --- | --- | --- | --- | --- |
|  | |  | | **Up to 12-week treatment (N = 1086)** | | | | |
|  |  | | <60 years (N = 567) | | ≥60 years (N = 519) | Statistic coefficients^a^ | P-value |  |
| Gender, N (%) female |  | | 385 (67.9) | | 360 (69.4) | χ^2^ = 0.269 | P = 0.604 |  |
| Education, mean (SD) years |  | | 11.5 (3.7) | | 6.5 (4.5) | t = 19.548 | **P < 0.001^b^** |  |
| Marital status, N (%) unmarried |  | | 179 (31.6) | | 147 (28.3) | χ^2^ = 1.359 | P = 0.244 |  |
| Living alone, N (%) |  | | 61 (10.8) | | 106 (20.4) | χ^2^ = 19.454 | **P < 0.001^b^** |  |
| Religious observance, N (%) |  | | 287 (50.6) | | 320 (61.7) | χ^2^ = 13.396 | **P < 0.001^b^** |  |
| Unemployed status, N (%) |  | | 156 (30.2) | | 160 (28.1) | χ^2^ = 0.614 | P = 0.433 |  |
| Monthly income, N (%) <2,000 USD |  | | 233 (41.1) | | 415 (80.0) | χ^2^ = 170.104 | **P < 0.001^b^** |  |
| Body mass index, mean (SD) kg/m^2^ |  | | 23.1 (3.4) | | 23.4 (3.0) | t = -1.643 | P = 0.101 |  |
| Major depressive disorder, N (%) |  | | 481 (84.8) | | 444 (85.5) | χ^2^ = 0.110 | P = 0.740 |  |
| Melancholic feature, N (%) |  | | 71 (12.5) | | 91 (17.5) | χ^2^ = 5.362 | **P = 0.021** |  |
| Atypical feature, N (%) |  | | 56 (9.9) | | 13 (2.5) | χ^2^ = 24.748 | **P < 0.001^b^** |  |
| Age at onset, mean (SD) years |  | | 41.6 (13.0) | | 63.1 (12.5) | t = -27.804 | **P < 0.001^b^** |  |
| Duration of illness, mean (SD) years |  | | 4.3 (7.1) | | 5.9 (10.7) | t = -2.815 | **P = 0.005** |  |
| Recurrent depression, N (%) |  | | 318 (56.1) | | 252 (48.6) | χ^2^ = 6.161 | **P = 0.013** |  |
| Number of depressive episodes, mean (SD) |  | | 1.2 (1.6) | | 0.9 (1.3) | t = 3.053 | **P = 0.002** |  |
| Duration of present episode, mean (SD) months |  | | 7.4 (10.2) | | 7.4 (10.6) | t = -0.133 | P = 0.894 |  |
| Family history of depression, N (%) |  | | 100 (17.6) | | 58 (11.2) | χ^2^ = 9.100 | **P = 0.003** |  |
| History of suicide attempt, N (%) |  | | 76 (13.4) | | 19 (3.7) | χ^2^ = 32.233 | **P < 0.001^b^** |  |
| Number of physical disorders, mean (SD) |  | | 1.2 (1.2) | | 2.1 (1.3) | t = -11.302 | **P < 0.001^b^** |  |
| Hamilton Depression Rating Scale |  | | 20.8 (4.2) | | 20.7 (4.1) | t = 0.201 | P = 0.841 |  |
| Hospital Anxiety & Depression Scale-anxiety subscale |  | | 12.4 (4.2) | | 11.2 (3.8) | t = 4.767 | **P < 0.001^b^** |  |
| EuroQol-5D |  | | 8.9 (1.5) | | 9.0 (1.6) | t = -1.431 | P = 0.153 |  |
| Social and Occupational Functional Assessment Scale |  | | 55.5 (7.3) | | 56.4 (7.6) | t = -1.892 | P = 0.059 |  |

^a^Independent two sample *t*-test or χ^2^ test, as appropriate.

^b^Values show statistical significance after Bonferroni correction.

| **Supplementary Table 2.**  Interaction effects of baseline serum 5-HT level and age as continuous variables on the 12-week remission rate | | | |
| --- | --- | --- | --- |
| Exposure |  | Interaction effect | |
|  |  | OR (95% CI) | P-value |
| All participants (N =1086) |  |  |  |
| 5-HT (increasing) × age (increasing) |  | 1.15 (1.06-1.25) | **0.001** |

^a^ All data were adjusted for education, living alone, religious observance, melancholic features, atypical features, age at onset, duration of the present episode, HAMD, HADS-A, and SOFAS.

| **Supplementary Table 3.**  Baseline characteristics based on initial antidepressant type in patients with depressive disorders | | | | | | | |  |
| --- | --- | --- | --- | --- | --- | --- | --- | --- |
|  | |  | | **Up to 12-week treatment (N = 1086)** | | | | |
|  |  | | SSRIs (N = 672) | | Non-SSRIs (N = 414) | Statistic coefficients^a^ | P-value |  |
| Age, mean (SD) years |  | | 56.1 (15.4) | | 58.3 (14.0) | t = -2.386 | **P = 0.017** |  |
| Gender, N (%) female |  | | 450 (67.0) | | 295 (71.3) | χ^2^ = 2.191 | P = 0.139 |  |
| Education, mean (SD) years |  | | 9.2 (4.8) | | 8.9 (4.8) | t = 1.232 | P = 0.218 |  |
| Marital status, N (%) unmarried |  | | 210 (31.3) | | 116 (28.0) | χ^2^ = 1.273 | P = 0.259 |  |
| Living alone, N (%) |  | | 100 (14.9) | | 67 (16.2) | χ^2^ = 0.334 | P = 0.563 |  |
| Religious observance, N (%) |  | | 370 (55.1) | | 237 (57.2) | χ^2^ = 0.497 | P = 0.481 |  |
| Unemployed status, N (%) |  | | 187 (27.8) | | 129 (31.2) | χ^2^ = 1.379 | P = 0.240 |  |
| Monthly income, N (%) <2,000 USD |  | | 392 (58.3) | | 256 (61.8) | χ^2^ = 1.306 | P = 0.253 |  |
| Body mass index, mean (SD) kg/m^2^ |  | | 23.2 (3.1) | | 23.3 (3.4) | t = -0.427 | P = 0.669 |  |
| Major depressive disorder, N (%) |  | | 564 (83.9) | | 361 (87.2) | χ^2^ = 2.169 | P = 0.141 |  |
| Melancholic feature, N (%) |  | | 96 (14.3) | | 66 (15.9) | χ^2^ = 0.554 | P = 0.457 |  |
| Atypical feature, N (%) |  | | 44 (6.5) | | 25 (6.0) | χ^2^ = 0.112 | P = 0.738 |  |
| Age at onset, mean (SD) years |  | | 1.5 (0.5) | | 1.5 (0.5) | t = 0.036 | P = 0.971 |  |
| Duration of illness, mean (SD) years |  | | 4.8 (8.9) | | 5.6 (9.2) | t = -1.435 | P = 0.152 |  |
| Recurrent depression, N (%) |  | | 345 (51.3) | | 225 (54.3) | χ^2^ = 0.930 | P = 0.335 |  |
| Number of depressive episodes, mean (SD) |  | | 1.0 (1.4) | | 1.2 (1.6) | t = -1.600 | P = 0.110 |  |
| Duration of present episode, mean (SD) months |  | | 7.0 (9.8) | | 8.0 (11.4) | t = -1.433 | P = 0.152 |  |
| Family history of depression, N (%) |  | | 98 (14.6) | | 60 (14.5) | χ^2^ = 0.002 | P = 0.967 |  |
| History of suicide attempt, N (%) |  | | 65 (9.7) | | 30 (7.2) | χ^2^ = 1.889 | P = 0.169 |  |
| Number of physical disorders, mean (SD) |  | | 1.5 (0.5) | | 1.5 (0.5) | t = -0.083 | P = 0.933 |  |
| Hamilton Depression Rating Scale |  | | 20.4 (4.0) | | 21.3 (4.4) | t = -3.512 | **P < 0.001^b^** |  |
| Hospital Anxiety & Depression Scale-anxiety subscale |  | | 12.0 (4.2) | | 11.6 (3.8) | t = 1.602 | P = 0.110 |  |
| EuroQol-5D |  | | 8.9 (1.5) | | 9.0 (1.5) | t = -1.994 | **P = 0.046** |  |
| Social and Occupational Functional Assessment Scale |  | | 56.7 (7.1) | | 54.7 (7.8) | t = 4.465 | **P < 0.001^b^** |  |

^a^Independent two sample *t*-test or χ^2^ test, as appropriate.

^b^Values show statistical significance after Bonferroni correction.

| **Supplementary Table 4.**  12-week remission rate, serum 5-HT level, and age based on type of initial antidepressant | | | | |
| --- | --- | --- | --- | --- |
| Exposure | **Up to 12-week treatment (N = 1086)** | | | |
|  | SSRIs (N = 672) | Non-SSRIs (N = 414) | Statistic coefficients^a^ | P-value |
| 12-week remission, N (%) | 315 (46.9) | 175 (42.3) | χ^2^ = 2.193 | 0.139 |
| Serum 5-HT (ng/mL) |  |  |  |  |
| Median (IQR) | 74.6 (68.4) | 69.8 (71.0) | U = 132488.000 | 0.188 |
| Mean (SD) | 81.5 (53.5) | 76.9 (51.0) | t = 1.401 | 0.161 |
| Age (y) |  |  |  |  |
| Median (IQR) | 58.0 (10.0) | 60.0 (17.0) | U = 129913.000 | 0.067 |
| Mean (SD) | 56.1 (15.4) | 58.3 (14.0) | t = -2.386 | **0.017** |

^a^Independent two-sample *t*-test or χ^2^ test, as appropriate.

| **Supplementary Table 5.**  Individual effects of baseline serum 5-HT level and age on 12-week remission based on type of initial antidepressant | | | | | | | |
| --- | --- | --- | --- | --- | --- | --- | --- |
| Exposure | Group | 12-week remission | | | | | |
|  |  | SSRIs (N = 672) | | | Non-SSRIs (N = 414) | | |
|  |  | No. (%) presence | OR (95% CI) | | No. (%) presence | OR (95% CI) | |
|  |  |  | Unadjusted | Adjusted^a^ |  | Unadjusted | Adjusted^a^ |
| Serum 5-HT | Lower | 149 (45.4) | 1.00 | 1.00 | 85 (39.5) | 1.00 | 1.00 |
|  | Higher | 166 (48.3) | 1.12 (0.83-1.52) | 1.10 (0.80-1.51) | 90 (45.2) | 1.26 (0.85-1.87) | 1.12 (0.74-1.68) |
| Serum 5-HT  (increasing) | N/A | N/A | 1.12 (0.97-1.30) | 1.14 (0.98-1.34) | N/A | 1.15 (0.94-1.41) | 1.10 (0.89-1.36) |
| Age | <60 | 162 (44.8) | 1.00 | 1.00 | 80 (39.0) | 1.00 | 1.00 |
|  | ≥60 | 153 (49.4) | 1.20 (0.89-1.63) | 1.01 (0.65-1.57) | 95 (45.5) | 1.30 (0.88-1.93) | 0.95 (0.54-1.69) |
| Age  (increasing) | N/A | N/A | **1.11 (1.00-1.22)^*^** | 1.16 (0.94-1.43) | N/A | 1.15 (1.00-1.32) | 1.09 (0.83-1.45) |

^a^Adjusted for education, living alone, religious observance, melancholic features, atypical features, age at onset, duration of present episode, HAMD, HADS-A, and SOFAS. ^*^P < 0.05.

| **Supplementary Table 6.**  Interaction effects of baseline serum 5-HT level and age as continuous variables on the 12-week remission rate based on the initial antidepressant type | | | |
| --- | --- | --- | --- |
| Exposure |  | Interaction effect | |
|  |  | OR (95% CI) | P-value |
| SSRIs (N = 672) |  |  |  |
| 5-HT (increasing) × age (increasing) |  | 1.19 (1.07-1.32) | **0.001** |
| Non-SSRIs (N = 414) |  |  |  |
| 5-HT (increasing) × age (increasing) |  | 1.09 (0.94-1.26) | 0.247 |

^a^ All data were adjusted for education, living alone, religious observance, melancholic features, atypical features, age at onset, duration of present episode, HAMD, HADS-A, and SOFAS

**
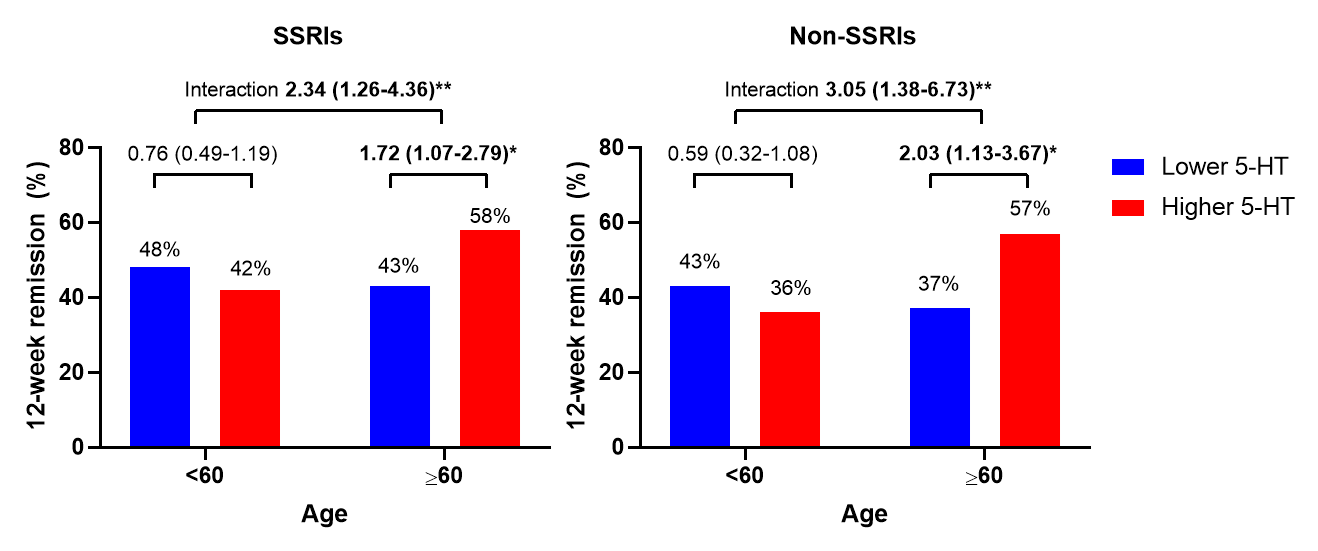
**

**Supplementary Figure 1.**

The 12-week remission rates according to baseline serum 5-HT level and age based on initial antidepressant type. Data are presented as odds ratios (95% confidence intervals) adjusted for educational level, living alone, religious observance, melancholic features, atypical features, age at onset, duration of present episode, HAMD score, HADS-A score, and SOFAS score. *P < 0.05; ******P < 0.01.
